# Competency-based compensation adjustment model: A new paradigm for clinical research coordinator merit advancement

**DOI:** 10.1017/cts.2026.10737

**Published:** 2026-03-31

**Authors:** Kate Marusina, Paige Kular, David Grigoryan, Brian Linhardt

**Affiliations:** 1 Clinical and Translational Science Center, UC Davis, USA; 2 Human Resources, UC Davis, Davis, USA

**Keywords:** clinical research, competency, compensation, clinical research coordinators, workforce

## Abstract

Attrition of experienced clinical research coordinators (CRCs) remains one of the most significant challenges to clinical and translational research. While multiple factors contribute to CRC retention, adequate compensation remains one of the most important. This manuscript describes a novel methodology for applying Joint Task Force (JTF) clinical research competencies as a guiding framework for salary adjustments via a pilot program for clinical research staff. This methodology can be adapted to a variety of institutional settings, especially in environments where opportunities for salary advancement are constrained by labor contracts. At UC Davis, CRC salary advancements are defined by contractual agreements with the Union of the Professional and Technical Employees, Research Support Professionals Unit (UPTE RX). While the union contract allows for periodic, across-the-board (all UPTE RX members) salary increases, it does not include clear provisions for merit-or competency-based increases. The CRC Equity Pathway is the first institutional compensation strategy that ties salary advancement to CRC competency, taking a significant step toward eliminating historical salary inequities. The pilot program described in this manuscript demonstrated that the CRC Equity Pathway is a viable mechanism for standardizing job descriptions around the JTF competencies, and for informing corresponding salary adjustments.

## Introduction

The 2023 report by the Society for Clinical Research Sites (SCRS) and the 2025 report by the Association for Clinical Research Professionals (ACRP) highlight the ongoing crisis of the global clinical research workforce [1,2]. These white papers agree on dire consequences for the clinical research enterprise if the shortage of qualified CRCs is not urgently addressed. Potential negative impacts include diminished quality of research operations, inconsistent data collection, prolonged timelines and missed enrollment goals. Key solutions identified include increasing awareness of the profession, expanding access to training and internships, creating clear advancement opportunities and strengthening site support [2]. Inadequate and stagnant salaries are widely recognized to be a major contributor to CRC attrition [3,4]. To improve retention via professional advancement opportunities, Duke University pioneered an evaluation methodology based on the JTF Competency Domains [5]. Duke’s extensive body of work included standardized job descriptions (JDs), streamlined role classifications, and a competency-based promotion process. A three-year analysis indicated a significant reduction in employee turnover (indicating improved retention) directly attributable to the implementation of a competency-based job framework with corresponding salary re-alignment [6].

The existing UC Davis CRC professional ladder is well defined and contains three classifications: Assistant CRC, CRC, and Senior CRC (Sr.CRC). Each classification has 25 salary steps. At the time of writing this manuscript, the hourly rate difference between consecutive steps ranged from $0.57 to $1.23 (Supplemental Figure 1). CRCs, along with other research support professionals, are represented by the UPTE-CWA 9119 (University Professional and Technical Employees, Communications Workers of America Local 9119), This labor union is specific to the University of California and Lawrence Berkeley National Laboratory. CRCs are members of one of the union units, the Research Professionals Unit (UPTE-RX), including over 5,700 research support professionals who provide highly specialized and complex scientific support to researchers [7]. The CRC yearly attrition rate at UC Davis ranges between 25 – 30%, in line with previously published turnover at Academic Medical Centers (AMCs) [6].

**Figure 1.**
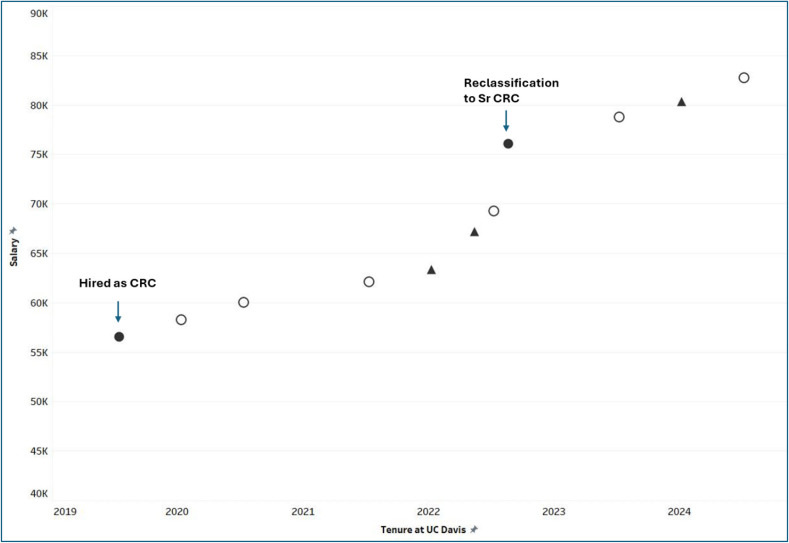
Hypothetical salary progression for an employee in the CRC ladder before initiation of the CRC equity pathway. At various timepoints, all members of the UPTE RX unit receive contractually-defined across-the-board salary increases. Open circles represent contractually-defined increases in which the value of each salary step increases by a certain percent (ranging 1.5–3.5%). Black triangles depict contractually-defined increases in which all UPTE RX members advance by one salary step. A more substantial increase may occur through re-classification (typically a 5% adjustment). Data for this figure is de-identified to protect individual anonymity.

The terms of the collective bargaining agreement (the union contract) have created unique challenges for salary advancement for CRCs. Over the years, the contract terms remained roughly the same, clearly specifying periodic across-the-board increases (typically one step, corresponding to approximately 2% increase), but not defining merit-or competency-based increases. Consequently, salaries for all CRCs advance uniformly and systematically, regardless of individual competency or years of service (Figure 1). Such mandated increases do not require updates in JDs. The only reliable mechanism for a substantial salary increase (5% or more) is an upward reclassification, from Assistant CRC to CRC and from CRC to Sr.CRC (Figure 1). While this option is often used as a retention strategy, it sometimes leads to premature promotions of CRCs who were not yet adequately prepared for the next level. In addition, this situation prompts high-performing employees to seek employment elsewhere, as their salaries became increasingly misaligned with their skills, abilities and job duties. A retention-focused methodology was therefore needed: one that would systematically capture accumulated competencies, skills, and abilities and translate them into standardized JDs with the corresponding salary increases, while retaining compliance with the union contractual provisions. Critically, the envisioned methodology would allow for either incremental “in range” salary progression (*within* the same classification) or for re-classification.

Duke’s Tier Advancement program became an inspiration for the CRC Equity Pathway at UC Davis. The CRC Equity Pathway represents the first successful institutional effort in the past decade to develop a centralized compensation adjustment methodology. The Pathway was developed by the workgroup consisting of UC Davis CTSC, Human Resources Compensation Unit, Employee and Labor Relations (ELR), UPTE, and clinical research supervisors. The resulting methodology allows for structured incremental adjustments in JDs correlative to acquired skills and competencies and for growth in salary unrestricted by a predetermined percentage. This approach eliminates the need for upward reclassification as a retention tool, rectifies historical salary inequities and lays clear framework for professional development. To achieve the Pathway’s goals, the workgroup developed: the Competency Assessment Survey (initially in REDCap, then in Trakstar software); a technology solution for Survey administration; a new administrative approval workflow, and a standardized approach for creation of JDs. The pilot program included eight employees from five Departments with the objectives of completing each implementation step, identifying challenges and barriers, and determining whether the CRC Equity Pathway presents a viable mechanism for competency-based salary increases.

## Materials and methods

The CRC Equity Pathway is uniquely tailored to the needs of AMCs. Development of the CRC Equity Pathway took over three years and involved multiple stakeholders across UC Davis, as listed above. The program is currently managed by the UC Davis CTSC and is comprised of the following key elements:

1. Competency-Assessment Survey.
2. Technology solution (Trakstar software) for data collection and analysis.
3. Administrative Review and Approval workflow.
4. Standardized approach to developing JDs.

Following the successful completion of the pilot described in this manuscript, ongoing efforts are focused on streamlining and improving the overall process flow.

**Competency-Assessment Survey.** UC Davis workgroup utilized the eight Joint Task Force (JTF) competency domains and the 48 competency statements as a basis for the CRC Competency Assessment. The JTF Competencies were developed as a unified, high-level set of standards for defining professional competence throughout the clinical research enterprise [8]. ACRP Core Competency Guidelines for Clinical Research Coordinators further defined these competencies by delineating proficiency levels and mapping them to specific CRC tasks [9]. In addition, the UC Davis workgroup drew upon the Title Picker, a REDCap-based tool developed by the Duke Office of Clinical Research (DOCR) as a part of their comprehensive Tier Advancement program [10]. The workgroup amalgamated these materials and adapted them to the UC Davis environment and terminology.

**Figure 2.**
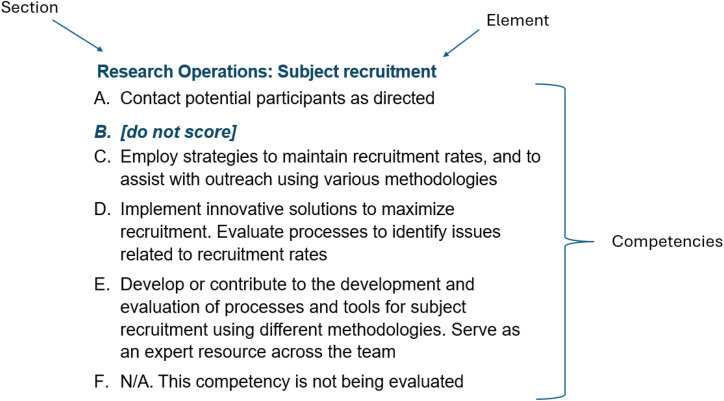
Structure of the UC Davis competency assessment. The assessment included seven sections: research operations, site and study management, data and informatics, leadership and professionalism, ethics and participant safety, communication and team science and scientific concepts and research design. Each section is further divided into elements, with each element containing a set of specific competencies. The complete content of the competency assessment is included in Supplemental Figure 2.

The final UC Davis CRC Competency Assessment survey consolidated eight domains into seven Sections. Similarly to the JTF Competencies, each Section is further subdivided into granular Elements (a total of 44 Elements). Each Element contains descriptions of competencies progressing from the entry-level (A) to the highest level (E) (Figure 2 and Supplemental Figure 2). Only one answer could be chosen for each Element. Therefore, each employee would provide 44 answers for the entirety of the Assessment. The pilot assessment was heavily weighted towards Research Operations, which accounted for 45% of all questions.

The option, “N/A-This competency is not being evaluated,” was included to acknowledge the diverse range of activities that coordinators perform depending on their team needs, organizational matrix, and types of studies. If the “N/A” answer is chosen, the corresponding Element is not scored. This feature is particularly important for CRCs with a limited scope of responsibilities, as it minimizes potential disadvantages in the final score calculation. Responses rated A–E are given a score (1 through 5). At the end of the survey, the score is averaged across all answered Elements, and the final score is used for determining the salary level (Supplemental Figure 1). Consequently, an employee performing multiple tasks at lower competency levels (as is typical for an Assistant CRC) will receive a lower overall score than an employee performing fewer tasks at higher competency levels (e.g., CRC or Sr.CRC). At UC Davis, this is viewed as a natural progression along the CRC career ladder.

In certain cases, the workgroup decided that an Element does not have a natural progression from A–E, and therefore, some of the answers and corresponding scores were omitted altogether (“do not score”).

It is important to note that the employees and supervisors were instructed to focus on competencies relevant to employee’s current work, rather than on overall competencies that an employee might possess (see below, Implementation Challenges and Lessons Learned). In addition, each supervisor composed a Guide to Competency Assessment, providing the interpretation of the questions in the context of their specific research unit.

The Competency Assessment Survey was initially programmed in REDCap and underwent multiple rounds of validation before being transferred into Trakstar Performance Management software.

**Technology Solution (Trakstar software) for Data Collection and Analysis.** Trakstar performance management software (Mitratech, Inc.) was selected for the production version of the Competency Assessment to reduce manual processing required by REDCap, and to leverage Trakstar’s automated workflows. Trakstar enables side-by-side comparison of employee and supervisor answers, provides dashboards for completion, and generates basic analytical reports. The software was significantly customized to accommodate the needs of this project. After an employee completes their self-assessment, the supervisor reviews the responses and adjusts as appropriate. Our experience indicates that this dual scoring approach is a critical feature for reducing assessment bias. When assessments are conducted solely by supervisors, results may lack full objectivity. Employees and supervisors receive Trakstar reports that provided side-by-side comparison supervisor and employee answers. The differences in scoring present an opportunity for discussion between supervisors and employees, followed by establishment of individual development plans aimed at achieving higher scores in the future. This discussion component was a required part of the CRC Equity Pathway Process and became an effective tool for clarifying discrepancies and fostering professional development.

**Figure 3.**
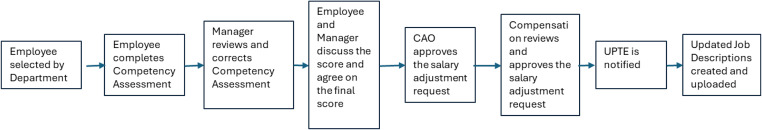
CRC equity review administrative process workflow (pilot).

**Administrative Review and Approval Workflow.** The workgroup created a new administrative workflow to accommodate the CRC Equity Pathway pilot (Figure 3). The pilot focused on Sr.CRC classification as the group most affected by historical salary inequities and in most need of retention. The pilot process began with the employee being selected by the supervisor and the Chief Administrative Officer (CAO). For the pilot, a wide discretion was given to Departments, who considered factors such as performance, longevity in the classification and length in current position etc. Once both the Sr.CRC and the supervisor completed the Competency Assessment and agreed on the final score, the supervisor submitted the data through a form transmitted to Compensation Analysts. After the final decision is reached, ELR notified the Union for approval. Upon receiving the UPTE approval, the new JDs based on the Competency Assessment were uploaded into the system of record (Job Builder), completing the process. The first pilot took approximately six months to complete, from October 2024–March 2025.

**Standardized Approach to Job Descriptions.** The completed Competency Assessments became a source of information for adjusting JDs in a standardized way. The answers for each employee were downloaded and categorized into four groups: A “Under Supervision”; B–D “Independently”; E “Resource to the team”; N/A “May contribute but not responsible for.”

The new layout helped to visually identify higher levels of responsibility and gave an option for a higher granularity of tasks. The pilot allowed for flexibility to include additional information or retain information from prior JDs. An example of a blended JD based on the Competency Assessment is shown in the Supplemental Figure 3.

## Results

### Employee selection and administrative process

The pilot CRC Equity Pathway focused on the Sr.CRC classification. This intentionally narrow scope allowed for focusing on a small defined pool of highly experienced staff, who are frequently perceived as being the most disadvantaged in compensation due to the contractually defined pay structure described above. Given a potentially significant financial impact, only a limited number of Departments chose to participate. Seven Sr.CRCs and five supervisors from five departments completed the entire pilot CRC Equity process. De-identified scores and pay increases for the pilot group are shown in the Supplemental Table 1. The median increase in hourly rate was $3.26. The median time for employee assessment was one day, and the median review time for supervisors was 26 days. The next step, approval by CAOs, varied significantly based on the Department, ranging from 3 days to 75 days. Once all requests were submitted to Compensation, the review process took about two months, with an additional month of review by UPTE. Overall, the pilot took 6 months to complete.

### Competency assessment survey results and interpretation

The distribution of answers of the 7 Sr.CRCs in the pilot group is shown in Table 1.

**Table 1.** Distribution of answers of the pilot group of Sr.CRCs. Percentages were calculated as follows: answers (A, B, C, D, E, N/A) for the seven employees were summed up for each Section and divided by the total number of collected answers for that Section. Raw data is not shown

Section	A (entry level)	B (under super-vision)	C (inde-pendent contributor)	D (Resource to the team; mentoring others)	E (Resource to the team; developing processes)	N/A (This competency is not being evaluated)
Research operations (%)	3	4	19	24	29	21
Site and study management (%)	2	3	24	10	35	27
Data and informatics (%)	0	0	10	14	45	31
Leadership and professionalism (%)	0	10	71	0	19	0
Ethics and participant safety (%)	0	0	29	0	57	14
Communications and team science (%)	0	0	7	0	93	0
Scientific concepts (%)	0	0	21	50	21	7

The distribution of answers for the first three sections (***Research Operations, Site/Study Management and Data and Informatics***) highlighted that the Sr.CRCs in the pilot group functioned as independent contributors (C) or served as resources for the entire team (D,E).

***Leadership and Professionalism*** competencies were the most challenging to translate into line items appropriate for CRC daily activities. This Section showed the least concordance between the employees and supervisor responses (data not shown).

In the ***Communication and Team Science*** and ***Ethics and Participant Safety*** Sections, the respondents overwhelmingly chose answer D, which indicated “the ability to train, support and guide study team members” and E, “serve as a resource for the team.” These choices were the most concordant between employees and supervisors.

The analysis of “N/A” answers (Table 1) revealed higher percentages of these answers in the following Sections: IP Management (15%), Data Management (21%), and Budgeting, Billing and Invoicing (10%). It is difficult to make any substantive conclusions from this data due to small sample and the specific construction of certain Elements which limited response options.

### Concordance of answers between Sr.CRCs and supervisors

**Figure 4.**
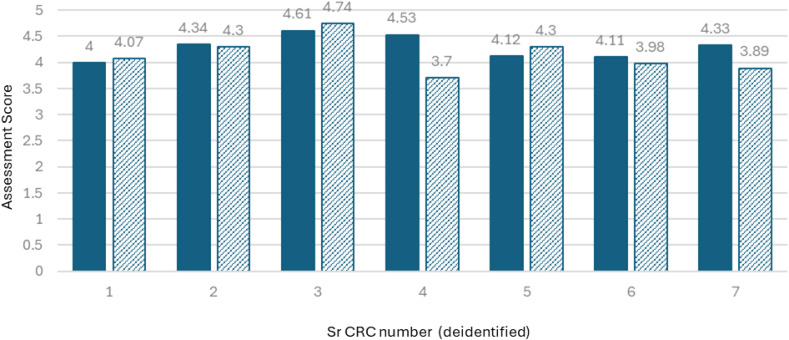
Comparison of the Sr.CRC self-assessment score and supervisor score. Seven employees in the pilot launch are numbered 1–7 to maintain anonymity. The employee score is shown as solid bars; supervisors’ scores are shown as striped bars.

Out of 308 data points (7 employees, 44 answers each), there were 48 discrepancies between employee and supervisor answers (85% concordance rate). Out of these discrepancies, employees rated themselves higher in 36 instances, and supervisors rated employees higher in 12. In most cases, employees chose the highest competency answer (E, serving as a resource for the team), whereas supervisors rated them as solid independent contributors (C). Sr.CRC#4 scored themselves 22% higher than the supervisor, and Sr.CRC#7 scored themselves 11% higher (Figure 4).

### Creating competency-based job descriptions

As described above, the answers from the competency assessment served as the foundation for competency-based JDs. Next, we had to align existing JDs with competency-based JDs. This translation was challenging, and matching the competency answers to existing JD sentences ended up being a laborious manual process. Old JDs emphasized specific activities, such as budgeting, start-up or sample collection, and were filled with redundant language, likely accumulated over years of copying and pasting. To ease the matching, we also divided the competency line items into sections, similar to old JDs. Despite the complexity of the alignment process, the resulting competency-based JDs were well received by the supervisors and HR analysts, and this translation was deemed a success. An example of the final competency-based JD is shown in Supplemental Figure 3.

## Discussion

The CRC Equity Pathway described in this manuscript demonstrated successful application of the JTF Competency Domains to an internal salary adjustment process. Our model was inspired by the previously published Title Picker, part of the Tier Advancement program implemented at the DOCR. The UC Davis CRC Equity Pathway model adapted the Title Picker principles to create a systematic methodology of capturing expansions in competencies with corresponding changes in job duties.

Our experience demonstrated that the published JTF Competencies only partially aligned with CRC functions at our University. The survey was heavily weighted towards ***Research Operations, Site/Study Management, and Data and Informatics,*** closely aligning with typical study management activities expected of a CRC at UC Davis. The answers trended at C,D, and E levels, reflecting the expected level of competency for Sr.CRCs. Similarly, ***Communication and Team Science*** and ***Ethics and Participant Safety*** were consistently scored at D and E level, highlighting the advanced role of a Sr.CRC within study teams, such as being a resource to junior members or streamlining project execution. However, we found translation of the JTF competencies to be particularly challenging for the Sections “***Leadership and Professionalism, ‘Ethical and Participant Safety Considerations***’ and “***Scientific Concepts and Research Design***.” Despite multiple attempts, the workgroup was able to create only a couple of Elements for each section. It is also possible that higher-level activities such as “lead a committee, task force, or ad hoc group,” “development of grant proposals” and “analyzing study results” may fall outside of scope of the Sr.CRC position at UC Davis. Other examples of competency-based CRC evaluations similarly found it necessary to adapt questions and metrics to reflect unique cultural contexts, local regulatory environment or patient-centric approaches [11]. We plan for additional revisions for these two sections in the future iterations of the CRC Competency Assessment to better reflect realistic opportunities for our employees. This focus will also help in translating competencies into JDs.

Launching a program of this scale and impact required a concerted effort of multiple individuals within UC Davis as previously noted. The following challenges particularly relevant to AMCs need to be taken into consideration when implementing similar salary adjustment initiatives.

1. ***Contractual environment.*** UC Davis CRCs are included in the UPTE and are School of Medicine employees. In other AMCs, the clinical research staff may be employed by state agencies, hospitals, schools of medicine, etc. Employment structure may define options for salary adjustments, e.g. whether merit-or competency-based increases are even available. In our case, the UPTE contract did not specifically address merit-or competency-based increases, and thus these were not established prior to this initiative. The workgroup included Union representatives from the very beginning, and a transparent, inclusive approach helped to ensure approval of the project. An important factor in the adoption of the CRC Equity Pathway was reaching a shared understanding that the results of the assessment and following salary adjustments constitute an entirely University-based process, not subject to the union grievance procedures.
2. ***Costs.*** The initial vision for the CRC Equity pathway included evaluation of all employees in the CRC ladder and implementing corresponding salary adjustments. Such implementation strategy would have resulted in approximately $1.2 M in salary increases. This was not met favorably by many departments. Because research is primarily soft funded, such increases would require re-negotiation of clinical trial contracts, or potential staff reduction. In the absence of centralized funding from the School of Medicine to subsidize salary increases, a compromise approach was rolled out. For the purposes of the pilot, Departments were given flexibility to nominate specific employees for the CRC Equity Pathway based on available funding and other department-specific considerations. While this approach may come across as inequitable, we believe that it was sufficient to introduce key concepts and achieve pilot results.
3. ***Understanding the assessment.*** A consistent challenge was ensuring that the assessment responses reflected current job responsibilities, rather than historically acquired competencies. For example, if the employee had prior experience in IRB submissions but was not currently tasked with the regulatory work, the correct answer should be “N/A – this competency is not being evaluated” (as noted earlier, “N/A” answers do not negatively impact overall scoring). Department leadership was in broad agreement with this approach, emphasizing that compensation should reflect how competently employees perform current job duties and that employees should not be paid for unused skills. While this distinction was sometimes difficult to convey, the analysis ultimately demonstrated overall significant concurrence between the employee and supervisor answers. In most cases, the final scores were agreed upon after a constructive discussion between the employee and supervisor. We believe that dual employee and supervisor assessments are necessary to avoid biases. One department experimented with conducting only supervisor-based evaluations, which revealed inconsistencies in scoring approaches. While “supervisor-only” scoring may help to prioritize candidates for the CRC Equity Pathway, the final process is less subjective if both employee and supervisor assessments are included and discussed.
4. ***Standardized approach to job descriptions.*** The HR Compensation wholeheartedly welcomed the standardization approach to ease the cross-department comparisons. Nevertheless, when first introduced, the concept of standardization of JDs received significant pushback from supervisors who felt that the activities performed by staff are too varied and largely dependent on types of studies and disease indication. In the end, the CTSC took the initiative to manually re-design each individual JD using the Competency Assessment answers while still allowing for the local language. Such a blended, hands-on approach proved to be necessary during the pilot phase of the program.

As a result of the CRC Equity Pathway pilot, seven employees received salary increases. The process was highly collaborative from start to finish. As of the time of this manuscript, UC Davis expanded the CRC Equity Process to include CRCs in addition to Sr.CRCs, and is evaluating options to roll out to all employees in the CRC category. We have also significantly shortened the process by applying lessons learned from the pilot group. The authors believe that the Competency Assessment process as a mechanism for salary advancement can be adapted by any AMC regardless of union environment.

## Supporting information

10.1017/cts.2026.10737.sm001 Marusina et al. supplementary material Marusina et al. supplementary material

## Supplementary material

The supplementary material for this article can be found at https://doi.org/10.1017/cts.2026.10737.
