# Supplementary material for: Competency-based compensation adjustment model: A new paradigm for clinical research coordinator merit advancement
Source: J Clin Transl Sci. 2026 Mar 31;10(1):e80. doi: 10.1017/cts.2026.10737 (PMC13126407; doi:10.1017/cts.2026.10737)
Supplement: Marusina et al. supplementary material [file S2059866126107377sup001.docx]

**Supplemental Materials**

**Supplemental Figure 1. Structure of the salary steps for the employees in the CRC Ladder as of 2024.** The salary corresponding to steps somewhat overlap as shown in boxes. The CRC Equity project correlated salary steps with the competency assessment score (discussed below).

**Competency Assessment**

In the next sections, you will be asked to select the level of job responsibility you have in multiple domains. You may mark only one response per box, so select the one that best represents the work you currently do or may be asked to do at any time. If you do not routinely carry out any of the responsibilities as part of your job, mark "N/A," which means that this is not a part of your job. Certain questions will have *[do not score]* answers. Do not choose these answers. This is done to maintain correct score calculations on the backend. Your supervisor will review your selections and discuss any adjustments with you.

# **Research Operations (Good Clinical Practice)**

## **Research Operations: Pre-screen participants**

1. Understand the purpose of eligibility requirements and choice of pre-screen activities
2. Determine whether or not a subject is eligible for study participation. Conduct pre-screening activities, as directed
3. Conduct pre-screening activities, independently
4. Provide support, mentorship and training to study team members to ensure accurate subject inclusion
5. Design and implement tools and templates to assist in ensuring compliance with study eligibility requirements
6. N/A. This competency is not being evaluated

## **Research Operations: Subject recruitment**

1. Contact potential participants as directed

#### [do not score]

1. Employ strategies to maintain recruitment rates, and to assist with outreach using various methodologies
2. Implement innovative solutions to maximize recruitment. Evaluate processes to identify issues related to recruitment rates
3. Develop or contribute to the development and evaluation of processes and tools for subject recruitment using different methodologies. Serve as an expert resource across the team
4. N/A. This competency is not being evaluated

## **Research Operations: Subject retention**

1. Maintain retention activities per protocol

#### [do not score]

1. Contribute to or implement innovative solutions to maximize retention
2. Evaluate processes to identify issues related to retention and propose solutions
3. Develop or contribute to the development and evaluation of processes and tools related to subject retention using different methodologies. Serve as an expert resource across the team
4. N/A. This competency is not being evaluated

## **Research Operations: Study level documentation**

1. Assist with maintenance of study level documentation.
2. Collaborate to maintain appropriate study-level documentation
3. Independently maintain appropriate study-level documentation
4. Provide support, mentorship and guidance to staff to maintain exemplary documentation
5. Develop or contribute to the development and evaluation of processes and tools for study level documentation
6. N/A. This competency is not being evaluated.

## **Research Operations: Delegation of Responsibilities**

1. Explain the purpose of a Delegation of Responsibilities Log and describe the roles of study team members. Explain the process for adding additional team members to the Delegation of Responsibilities log and the training documentation required.
2. Complete Delegation of Responsibilities log, under supervision
3. Independently maintain Delegation of Responsibilities log
4. Provide support, mentorship and guidance to staff to maintain Delegation of Responsibilities log
5. Develop or contribute to the development of processes for designating staff roles and for maintaining Delegation of Responsibilities logs.
6. N/A. This competency is not being evaluated.

## **Research Operations: Subject level documentation (source documents, participant communication, etc)**

1. Understand the requirements for subject-level documentation
2. Maintain subject level documentation, under direction
3. Maintain subject level documentation, independently
4. Provide support, mentorship and training to study team members who maintain subject level documentation, including documentation in the EMR
5. Develop or contribute to the development and evaluation of best practices, processes and tools for subject level documentation
6. N/A. This competency is not being evaluated

## **Research Operations: Consent procedures**

1. Explain the purpose of an Informed Consent as part of subject safety. Describe the required elements of an informed consent form and explain the essential elements of the informed consent process. Discuss important factors influencing the evolution of informed consent requirements. Provide an assisting role in a consent process
2. Develop and review draft informed consent documents in compliance with regulatory requirements and GCPs. Demonstrate the ability to conduct and document an informed consent discussion as directed
3. Develop consent documents, conduct and document consent for participants independently and/or provide training to others in these tasks
4. Provide support, mentorship and guidance to study team members who conduct and document consent for participants
5. Develop tools, resources, and best practices to enhance the informed consent and subject education processes to further enhance subject protections in the team
6. N/A. This competency is not being evaluated.

## **Research Operations: Prepare and conduct study visits**

1. Understanding necessary components for a study visit based on protocol requirements
2. Conduct study visits, as directed
3. Conduct study visits independently
4. Train others to conduct and document visits and protocol-specific testing/interviews
5. Develop or contribute to the development and evaluation of best practices, processes and tools for conducting study visits
6. N/A. This competency is not being evaluated

## **Research Operations: Stewardship of Supplies & Equipment**

1. Identify all study supplies & equipment needed for clinical study execution within a given clinical study.
2. Assess the study supply & equipment requirements and ensure they are available and appropriate for high quality study conduct, as directed
3. Maintain level of inventory necessary to conduct study activities and verify that all supplies & equipment are maintained in compliance with protocol requirements and local regulations, independently

#### [do not score]

1. Develop study supply specification tools / job aids and support study team members in determining study supply & equipment requirements. Establish processes for managing supply inventory.
2. N/A. This competency is not being evaluated

## **Research Operations: Specimen collection and preparation**

1. Assist with collection, preparation, processing, labeling and shipping of research specimens
2. Collect, prepare, process, ship, and maintain inventory of research specimens as directed
3. Collect, prepare, process, ship, and maintain inventory of research specimens independently
4. Provide support, mentorship and training to staff involved in research specimens management
5. Develop or contribute to the development, evaluation and optimization of workflows and/or logistical management for research specimens
6. N/A This competency is not being evaluated.

## **Research Operations: Investigational Products (IP)**

1. Understand the investigational product (IP) workflow for its handling and/or accountability
2. Maintain IP workflows and/or accountability under direction
3. Implement and maintain IP workflows and /or accountability independently
4. *[do not score]*
5. Develop or contribute to the development of processes, tools and training to ensure study team members are compliant in maintaining study accountability of IP.
6. N/A. This competency is not being evaluated.

## **Research Operations: Investigational Products (IP) - subject instruction**

#### [do not score]

1. Instruct the subject on use of investigational product, under supervision
2. Instruct the subject on use of investigational product independently

#### [do not score]

1. Develop or contribute to the development of processes, tools and training to enhance the process of instructing subjects on the use of investigational products.
2. N/A. This competency is not being evaluated.

## **Research Operations: Monitoring**

1. Understand the principles of the monitoring process including the role of the site, PI and CRC, and related documentation necessary for effective study monitoring.
2. Assist in preparation of documentation for monitor review as directed
3. Independently prepare and manage a monitoring visit including appropriate preparations, support, documentation and follow up

#### [do not score]

1. Develop or contribute to the development and evaluation of best practices, processes and tools related to monitoring. Serve as an expert resource when addressing and correcting findings
2. N/A. This competency is not being evaluated

## **Research Operations: Audits (examples: IRB, FDA, Sponsor)**

1. Understand the purpose of and process for different types of clinical study audits and inspections b. Prepare and assist with study audits as directed
2. Independently prepare for study audits
3. Provide direction to study teams with preparation for study audit visits. Address and correct audit findings.
4. Develop or contribute to the development of processes, tools and training to support the preparation, participation, documentation and follow-up actions and process improvement for clinical audits or inspections. Serve as an expert resource when addressing and correcting findings.
5. N/A. This competency is not being evaluated.

## **Research Operations: Adverse Event Data Collection**

1. Understand principles of safety monitoring including adverse event data identification, collection, and classification. Define all types of adverse events / effects (e.g., AE, SAE, Events of Interest, UADE, SUSAR) and the process for capturing, assessing, determining causal relationship of the event to the investigational product, investigator oversight, reporting and following up on adverse events.

#### [do not score]

1. Identify, classify and document adverse event information under supervision
2. Identify a full range of subject safety issues, and demonstrate the ability to document, report and follow up on these issues.
3. Develop or contribute to the development of best practices, processes and tools to support study team members in recognizing when subject safety issues have occurred and how to best manage these.
4. N/A. This competency is not being evaluated.

## **Research Operations: Adverse Event Reporting (to IRB, Sponsor, FDA)**

1. Understand adverse event reporting rules (timelines, tracking and forms)
2. Complete and submit Adverse Events Reports, according to institution and sponsor requirements under direction
3. Complete and submit AE Reports, according to institution and sponsor requirements independently
4. Provide mentorship and assistance to team members with submission and completion of AE Reports, according to institution and sponsor- specific requirements (timelines and forms)
5. Develop or contribute to the development of best practices, processes and tools for AE reporting.
6. N/A. This competency is not being evaluated.

## **Research Operations: IRB Initial Submissions**

1. Explain the Institutional Review Board (IRB) review, approval and reporting requirements for the site. Describe the differences between central and local IRB requirements and which type governs the conduct of a given clinical study. Understand requirements for the Initial IRB Application form and the required documents
2. Draft IRB-Required documents (i.e. informed consent) under direction
3. Review and complete the initial IRB Application form and required documents independently

#### [do not score]

1. Guide junior staff in the completion of the initial IRB submission. Develop or contribute to the development of best practices, processes and tools to assist with the initial IRB submission
2. N/A. This competency is not being evaluated.

## **Research Operations: IRB Post Approval Review - Continuing Review**

1. Understand requirements for the Post Approval Review - Continuing Review section and the forms
2. Draft IRB-Required documents for Continuing review under direction.
3. Prepare and submit the Continuing Review independently
4. [do not score]
5. Provide mentorship to junior staff. Develop or contribute to the development of best practices, processes and tools pertinent to the Continuing Review
6. N/A. This competency is not being evaluated.

## **Research Operations: IRB Post Approval Review - Modification**

1. Understand IRB reporting requirements for modifications and specific forms
2. Draft IRB-Required documents for modification under direction
3. Prepare and submit the modifications independently

#### [do not score]

1. Provide mentorship to junior staff. Develop or contribute to the development of best practices, processes and tools pertinent to the reporting of protocol modifications
2. N/A. This competency is not being evaluated.

## **Research Operations: Corrective and Prevention Action (CAPA)**

1. Understand the principles of Corrective and Prevention Action (CAPA) Plans, when they are needed and the process for completing, managing and following up on CAPA plans
2. Create, submit and implement CAPA plan under direction
3. Create, submit and implement CAPA independently

#### [do not score]

1. Guide study team members in CAPA assessments (including risk assessments, root cause analyses, etc.) and CAPA plan documentation. Lead CAPA activities to ensure appropriate documentation, follow through and prevention of future issues.
2. N/A. This competency is not being evaluated.

**Site and Study Management**

**Site and Study Management: Determining Feasibility of the site participation**

1. Understand the feasibility assessment process, including the facilities and equipment needed for a given clinical study.
2. *[do not score]*
3. Follow appropriate steps, gathering information and resources needed to conduct a thorough and appropriate feasibility assessment for a clinical study at UC Davis, under direction
4. Make recommendations to investigators and study teams regarding feasibility of the study implementation, including alternative solutions to accomplish goals. Conduct thorough feasibility assessments.
5. Develop or contribute to development of best practices, processes and tools for enhancing the study feasibility assessment process. Evaluate historical performance metrics and incorporate findings into future assessments to ensure more predictable enrollment performance and successful study execution. Proactively provide recommendations to sponsors/CROs/PI to improve protocol feasibility.
6. N/A. This competency is not being evaluated.

## **Site and Study Management: Budget and Coverage Analysis**

#### [do not score]

1. Explain the elements of a study budget as it relates to execution of a protocol.
2. Demonstrate the ability to assess a protocol and study plan to contribute to the development of a Coverage Analysis and study budget.

#### [do not score]

1. Develop budget and coverage analysis documents. Negotiate budget and payment terms and submit to contracts. Process budget amendments and ensure all documents are harmonized. Reconcile budget and actuals provided in faculty reports and identify deficiencies. Develop or support the development of tools and training to aid in the development of study budgets.
2. N/A. This competency is not being evaluated.

## **Site and Study Management: Research Billing Review**

1. Correctly associate participants, visits and orders with a study in EMR.

#### [do not score]

1. Able to review billing report and determine routing of charges, coordinate with IT/Billing to resolve issues as necessary

#### [do not score]

1. Provide expert guidance/oversight to study team members to ensure participant care expenses have been set up correctly and that financial charges/expenses route in a timely manner
2. N/A. This competency is not being evaluated.

## **Site and Study Management: Research Invoicing**

1. Understand the principles of sponsor invoicing
2. Be able to recognize and identify an invoiceable event during study performance and communicate with a relevant team, under direction.
3. Be able to recognize and identify an invoiceable event during study performance and communicate with a relevant team, independently

#### d.[do not score]

1. e. Develop or contribute to development of best practices and workflows to ensure accurate tracking of invoiceable items and timely invoicing
2. N/A. This competency is not being evaluated.

## **Site and Study Management: Managing PHI**

### Understand principles and necessary steps for accessing and disclosing identifiable health information in EMR. Explain the difference between requests for medical records and release or disclosure of medical records. Describe the institutional resources available to support the medical records collection and release processes.

### [do not score]

### Appropriately access PHI under federal, local and institutional privacy regulations / guidelines. Obtain and provide necessary disclosures (e.g., HIPAA waivers, Quick disclosures in EMR, etc.).

### [do not score]

### Develop or contribute to the development of policies, procedures and tools to ensure compliant collection or release of medical records of subjects under federal, local and institutional privacy regulations / guidelines (i.e. "Preparatory for Research", IRB waivers)

### N/A. This competency is not being evaluated.

## **Site and Study Management: Site Initiation Visits**

1. Describe the purpose of a site initiation visit and explain what is involved in the site initiation / start-up process for a clinical study. Prepare items for site initiation visits (i.e. scheduling, signatures), as directed
2. Participate in site initiation visits, as a team member

#### [do not score]

1. Lead site initiation activities and serve as an institutional resource to the study visitors
2. Develop or contribute to the development of processes, tools and training to guide study team members through the site selection process. Evaluate site selection performance metrics and provide recommendations for enhancing site practices to improve the site selection success rate. Contribute to development of best practices and workflows for site initiation visits
3. N/A. This competency is not being evaluated.

## **Site and Study Management: Study Closeout**

#### [do not score]

1. Understand the site and study closeout procedures, including related documents retention / archiving requirements. Participate in study closeout visits and perform closeout activities under direct supervision.
2. Take part in the study closeout and document storage activities
3. Lead study closeout procedures for a variety of clinical studies including the accurate completion, filing and storage of essential study documents.
4. Develop or contribute to the development of processes, tools and training for study closeout and document management activities.
5. N/A. This competency is not being evaluated.

## **Site and Study Management: Standard Operating Procedures/Work Instructions**

1. *[do not score]*

### Able to understand and follow the existing SOPs/WIs

1. Able to revise SOPs /WIs as needed, independently

#### [do not score]

1. Develop or contribute to the development of SOPs/WIs and provide training to the team members
2. N/A. This competency is not being evaluated.

## **Site and Study Management: Conflict of Interest (COI)**

1. Explain what constitutes conflict of interest in a clinical study and provide an example. Explain the process and forms associated with financial disclosure during clinical studies.
2. Submit COI for a study and maintain the logs, under supervision
3. Maintain COI disclosures submissions and logs, independently

#### [do not score]

1. Serve as a resource to the team in fostering a better understanding of conflicts of interest. Develop or contribute to development of best practices, processes and tools for managing conflict of interests.
2. N/A. This competency is not being evaluated.

**Data Management**

**Data and Informatics: Creation of Source Documents**

1. Understand the purpose and structure of source documents. Describe how to capture study visit data using different types of source documents.
2. Design and create Source Documents consistent with the protocol and CRFs, under direction. Demonstrate the ability to apply ALCOA-C practices to the collection of data in a given clinical study.
3. Design and create Source Documents consistent with the protocol and CRFs, independently. Able to collect, verify and report data via source documentation to enable source data verification by monitors.

#### [do not score]

1. Guide study team members on best source documentation and data collection practices. Develop or contribute to development of best practices, processes and tools pertinent to Source Documents
2. N/A. This competency is not being evaluated.

**Data and Informatics: Data Collection, including Electronic Data Collection (EDC) Systems and Case Report Forms (CRF)**

### Understand the purpose of Electronic Data Capture (EDC) systems, technologies, and software necessary for study operations

1. Enter data into Case Report Forms (CRFs and eCRFs) accurately and according to protocol, under direction
2. Manage submission of data into Case Report Forms (CRFs and eCRFs) accurately and according to protocol, independently
3. Train junior staff in use of technologies and software, and in data entry into ECRFs. Detect issues related to data capture, data collection or management and suggest solutions.
4. Independently design and create new data collection instruments, tools, job aids and resources. Select methods of data capture with complete understanding of advantages and disadvantages of each.
5. N/A. This competency is not being evaluated.

## **Data and Informatics: Data Flow**

1. Evaluate areas of vulnerability for data flow or data collection and create mitigation plans for potential issues. Design data flow plans that include data capture, storage, data management, quality and preparation for analysis.
2. N/A. This competency is not being evaluated.

## **Data and Informatics: Data Corrections/Queries**

1. Explain the process of reviewing and resolving queries and list the benefits and importance of timely query resolution.
2. Identify and resolve data correction/queries, under direction
3. Independently identify and promptly address data correction/queries
4. Provide mentorship to junior staff on process for data correction/query resolution
5. Develop or contribute to best practices, processes and tools for queries resolution and proactively provide recommendations to study team and sponsor/CRO personnel on strategies to minimize query generation.
6. N/A. This competency is not being evaluated.

## **Data and Informatics: Data Quality Assurance (QA)**

### Develop or contribute to the development of processes and/tools to ensure security of data is maintained and accessed only by authorized personnel. Contribute to data quality assurance systems for research data; ensure that data QA SOPs are updated and followed by study teams. Implement data quality assurance systems across multiple studies, or study team. Recognize trends; escalate as necessary. Provide mentorship to junior staff

1. N/A. This competency is not being evaluated.

## **Data and Informatics: Data Contracts and Agreements (Data Use Agreement, Data Transfer Agreement, etc.)**

1. Recognize, provide input and assemble the necessary parties to ensure that all data agreements are in place
2. N/A. This competency is not being evaluated.

# **Ethical and Participant Safety Concerns**

## **Ethics and Participant Safety: Clinical vs Research Procedures**

1. Understand the difference between standard of care and clinical study activities and describe why it is important to make this distinction for the purposes of subject education, protocol compliance, financial billing and other facets as related to patient care and clinical study conduct.
2. Implement workflows for differentiating and managing clinical and research procedures under direction
3. Implement workflows independently

#### [do not score]

1. Serve as a resource to the team in fostering a better understanding of the important distinction between standard-of-care and research study activities. Develop or contribute to development of best practices, processes and tools for managing clinical and research procedures
2. N/A. This competency is not being evaluated.

## **Ethics and Participant Safety: Vulnerable populations**

1. Define ethical and regulatory considerations for the inclusion of vulnerable populations in clinical studies, and specifically with safeguards needed when conducting research with vulnerable populations.
2. Apply appropriate safeguards for the inclusion of vulnerable subjects into clinical studies. Participate in research activities involving vulnerable populations, under direction
3. Participate in research activities involving vulnerable populations, independently

#### [do not score]

1. Serve as a resource to the team in fostering a better understanding of research with vulnerable populations. Develop or contribute to development of best practices, processes and tools for integrating vulnerable populations in research
2. N/A. This competency is not being evaluated.

# **Scientific Concepts and Research Design**

## **Scientific Concepts: Research Design**

1. [do not select]
2. Identify and explain the study hypotheses, study objectives and endpoints for a variety of clinical studies.
3. Demonstrate an understanding of how study endpoints relate to the study procedures and data collection requirements necessary to prove the hypotheses for a variety of clinical studies.
4. Analyze protocols to identify study endpoints and outcomes. Translate the protocol priorities into a study implementation plan to ensure study objectives are achieved in a timely and compliant manner.
5. Develop or contribute to development of grant proposals or clinical research protocols. Assess and determine solutions for potential operational shortcomings of proposals and protocols.
6. N/A. This competency is not being evaluated.

## **Scientific Concepts: Study Results**

#### [do not score]

1. *[do not score]*
2. Locate and describe published results of a clinical trial.
3. Demonstrate the ability to read and interpret clinical study results. Describe situations in which the published study results did not support the study objectives, what contributed to this outcome and how this relates to proper study execution.
4. Analyze completed study results for development of recommendations to enhance quality outcomes.
5. N/A. This competency is not being evaluated.

# **Leadership and Professionalism**

## **Leadership and Professionalism: Apply Principles of Leadership**

#### [do not score]

1. Demonstrate development in leadership skills through contributions to the team's projects (i.e. process improvement)
2. Take part in a committee, task force, or ad hoc group. Or participate in scientific presentations and publications.

#### [do not score]

1. Lead a committee, task force, or ad hoc group. Or lead or initiate scientific or programmatic presentations and publications. Provide significant contribution and influence upon operational activities or productivity of the team.
2. N/A. This competency is not being evaluated.

## **Leadership and Professionalism: Guidelines and Code of Ethics**

### *[do not score]*

1. Explain professional guidelines and code of ethics as they apply to the role of the CRC.
2. Demonstrate the ability to apply professional guidelines and codes of ethics to enhance clinical study execution.

#### [do not score]

1. Develop or contribute to development of SOPs, guidelines, work instructions and training procedures and materials to convey the professional guidelines and codes of ethics for study team members to enhance the ethical and quality conduct of studies at the site.
2. N/A. This competency is not being evaluated.

## **Leadership and Professionalism: Cultural Diversity**

### Understand the concept of regional diversity and cultural competency. Cite examples of the diversity within the site's locale. Understand the importance of these concepts within overall clinical study design and implementation.

#### [do not score]

1. Demonstrate an understanding of the effect that regional diversity has on a site as well as overall clinical study recruitment and study results. Demonstrate the ability to incorporate cultural competency within a site's processes to improve communication and study engagement with diverse patient populations.

#### [do not score]

1. Develop or contribute to implementing processes which enhance cultural competency within a site resulting in a site's improved clinical study communication and engagement for study participation. Guide and support study team members with regard to the development and implementation of cultural competency procedures.
2. N/A. This competency is not being evaluated.

# **Communication and Team Science**

**Communication and Team Science: Communication with Stakeholders**

1. Understand the relationship between Sponsor, CRO and clinical research site personnel, trial participants and their family members, the subjects' Primary Care Physicians (PCPs) or treating physicians including the basics of appropriate communication chains of command. Describe the general types of correspondence that take place during a study and provide examples of situations requiring expedited communication. Able to clearly communicate (orally and in writing) with relevant stakeholders (participants, PI etc)

#### [do not score]

1. Understands when the sponsor/CRO needs to be alerted to a concern or when questions should be directed to the sponsor/CRO vs. the internal site study team. Able to escalate issues appropriately

#### [do not score]

1. Demonstrate the ability to train, support and guide study team members on communication practices amongst different stakeholders. Mentors others on effective communication and problem resolution.
2. N/A. This competency is not being evaluated.

## **Communication and Team Science: Teamwork**

1. Participate in study team meetings. Respond to routine questions related to study protocol and refer more complex questions to others as appropriate. Communicate and coordinate with other study personnel as required for study implementation and routine problem resolution.

#### [do not score]

1. Prepare for and lead team meetings. Recognize when others need to be brought into the conversation and escalate appropriately. Contribute to multi-disciplinary or inter-professional research teams

#### [do not score]

1. Demonstrate the ability to lead and collaborate across multi-disciplinary and inter-professional research teams. Contribute to process improvement in team communication or mentor junior staff to improve ability to participate in team efforts.
2. N/A. This competency is not being evaluated.

**Supplemental Figure 2 . The entire Competency Assessment**

| **Sr CRC number (de-identified)** | **Current Salary Step** | **Competency Assessment Score** | **Requested Salary Step** | **Increase in pay, hourly** |
| --- | --- | --- | --- | --- |
| 1 | 6 | 4.07 | 9 | $2.63 |
| 2 | 4 | 4.3 | 10 | $5.21 |
| 3 | 12 | 4.7 | 18 | $6.09 |
| 4 | 2 | 3.7 | 5 | $2.44 |
| 5 | 8 | 4.3 | 12 | $3.68 |
| 6 | 7 | 3.98 | 8 | $0.87 |
| 7 | 2 | 3.89 | 6 | $3.26 |

**Supplemental Table 1. Competency Assessment scores vis-à-vis Salary increases**. The table shows de-identified scores of Sr. CRCs who completed the entire Equity Pathway. The table compares salary steps of employees when entering the CRC Competency pathway, proposed salary steps based on the scoring and the hourly rate increase generated by the assessment.

Supplemental Figure 3

An example of a blended Competency-based Job Description. Text in blue represents answers from the Competency Assessment. Answers in black were retained from the original job description. Adapted for the publication.

| **Function** | **Duties** |
| --- | --- |
| Site and Study Management and Research Operations | **Independently**   - Serve as the lead coordinator for large, dual site study with complex ancillary study requirements. Act as main point of contact for all study related concerns. - Conduct and document Informed Consent for study participants. Ensure all necessary signatures and dates are on the informed consent (ICF) and HIPAA. - Monitor and respond to inquiries on the study phone line. - Submit and implement corrective and preventative action plans (CAPA) - Maintain retention activities - Schedule participants and conduct study visits - Maintain compliant EMR records pertinent to research   **Serve as a resource for the entire team:**   - Provide support, mentorship and training to study team members to ensure accurate subject inclusion - Develop or contribute to the development, evaluation and optimization of workflows and/or logistical management for research specimens - Develop and evaluate processes and tools for subject and study level documentation - Develop or contribute to the development of policies, procedures, and tools to ensure compliant collection or release of medical records of subjects under federal, local and institutional privacy regulations / guidelines (i.e. "Preparatory for Research", IRB waivers) - Develop tools, resources, and best practices to enhance the informed consent and subject education processes to further enhance subject protections in the team - Develop study supply specification tools / job aids and support study team members in determining study supply & equipment requirements. Establish processes for managing supply inventory - Develop or contribute to the development, evaluation and optimization of workflows and/or logistical management for research specimens - Serve as a resource to the team in fostering a better understanding of the important distinction between standard-of-care and research study activities. Develop or contribute to development of best practices, processes and tools for managing clinical and research procedures - Serve as a resource to the team in fostering a better understanding of research with vulnerable populations. Develop or contribute to development of best practices, processes and tools for integrating vulnerable populations in research - Develop or contribute to development of SOPs, guidelines, work instructions and training procedures and materials to convey the professional guidelines and codes of ethics for study team members to enhance the ethical and quality conduct of studies at the site.   **May contribute but not responsible for**:   - Design of research protocols - Developing budgets and coverage analysis - Research invoicing - Investigational Drug Management and Accountability - Participation in Audits or Monitoring visits - Site Initiating Visits - Site Close outs - Study Feasibility assessments - Preparation of IRB Initial Submissions, Continuing Reviews, and Modifications - Submission of Conflict-of-Interest Disclosures - Research Billing Reviews |
| Data Management | **Independently:**   - Manage Data submission in Case report forms and answer queries. - Design and create new data collection instruments, tools, job aids and resources. - Complete timely and accurate data entry and maintenance of assigned studies in clinical trials management system.   **Serve as a resource for the entire team:**   - Generate Data Flow Plans - Oversee tracking systems, ensuring all staff utilize systems accurately, manage tracking system reports and review with staff to ensure timely and accurate submissions of data. - Guide study team members on best source documentation and data collection practices. - Develop or contribute to development of best practices, processes and tools pertinent to Source Documents |
| Leadership, Professionalism and Communication | - Provide leadership and mentorship to research coordinators throughout network sites - Participate in continuing education activities and trainings relevant to pediatric emergency medicine clinical trials. - Contribute and/or provide analytical support to process improvement. - Lead and collaborate across multi-disciplinary and inter-professional research teams. Contribute to process improvement in team communication or mentor junior staff to improve ability to participate in team efforts. - Develop or contribute to development of SOPs, guidelines, work instructions and training procedures and materials. - Mentor team members and be a resource in department-specific compliance and training documents and oversee staff compliance via training and certifications. - Appropriately alert sponsor/CRO to a concern or direct questions to the internal site study team. Escalate issues appropriately. - Instruct faculty on use of resources and with changes in research and university requirements. - Work with the department research leadership team on strategic planning for the research program, including staffing, research agenda development, funding projections and problem resolution. - Take part in a committee, task force, or ad hoc group. Or participate in scientific presentations and publications. - Provide oversight of research staff, student research assistants and volunteers who recruit patients in the Emergency Department and perform research study activities. - Coordinate research meetings and trainings and acts as resource for lower-level clinical research coordinators and other support staff. |
